# Identification of Receptor Binding Proteins in Flagellotropic *Agrobacterium* Phage 7-7-1

**DOI:** 10.3390/v13071267

**Published:** 2021-06-29

**Authors:** Floricel Gonzalez, Birgit E. Scharf

**Affiliations:** Department of Biological Sciences, Virginia Tech, Life Sciences 1, Blacksburg, VA 24061, USA; floric1@vt.edu

**Keywords:** depolymerase, flagella, growth inhibition, host, LPS, phage, RBP

## Abstract

The rapid discovery of new and diverse bacteriophages has driven the innovation of approaches aimed at detailing interactions with their bacterial hosts. Previous studies on receptor binding proteins (RBPs) mainly relied on their identification in silico and are based on similarities to well-characterized systems. Thus, novel phage RBPs unlike those currently annotated in genomic and proteomic databases remain largely undiscovered. In this study, we employed a screen to identify RBPs in flagellotropic *Agrobacterium* phage 7-7-1. Flagellotropic phages utilize bacterial flagella as receptors. The screen identified three candidate RBPs, Gp4, Gp102, and Gp44. Homology modelling predicted that Gp4 is a trimeric, tail associated protein with a central β-barrel, while the structure and function of Gp102 and Gp44 are less obvious. Studies with purified Gp4_1-247_ confirmed its ability to bind and interact with host cells, highlighting the robustness of the RBP screen. We also discovered that Gp4_1-247_ inhibits the growth of host cells in a motility and lipopolysaccharide (LPS) dependent fashion. Hence, our results suggest interactions between Gp4_1-247_, rotating flagellar filaments and host glycans to inhibit host cell growth, which presents an impactful and intriguing focus for future studies.

## 1. Introduction

Bacteriophage centered research and applications have boomed in recent years due to greater numbers of antibiotic resistant bacterial infections and thus the growing need for antibiotic alternatives [1,2,3]. Consequently, phage discovery initiatives have increased to build both public and private phage repositories, which resulted in a ten-fold rise in phage genomes deposited in publicly available databases in a span of 20 years [3]. Detailed investigations of phage infection mechanisms have also grown in number, albeit at a much slower pace. These efforts are hindered by two conflicting factors: (1) the need for rapid discovery of phages with specific host ranges for phage therapy applications versus (2) the time-consuming experimental characterization of phage gene product function. Thus, phage characterization with the end goal of rapidly producing phage therapies primarily focuses on determining host range, whole phage genome sequencing, morphological characterization, measurements of killing efficiencies in host planktonic or biofilm states, and, less commonly, identification of cell surface receptors [1,2,4,5].

A focus on interactions taking place during viral entry (i.e., phage binding and interactions with receptors) can yield a plethora of information, including the underlying determinants of host range, potential mechanisms of phage resistance, and evolutionary tradeoffs following phage treatment. Methods for the rapid detection of phage binding to host cells and identification of receptors have been developed and improved upon to make the approaches high throughput [5,6,7]. However, phage-host interactions during viral entry are typically investigated unilaterally, largely focusing on the bacterial hosts. Such studies have identified outer membrane proteins, efflux pumps, capsular polysaccharide (CPS), lipopolysaccharide (LPS), pili, and flagella as receptors used by bacteriophages [8,9,10,11,12,13,14]. Inarguably, a holistic understanding of the mechanisms underlying phage entry entails equally detailed analyses of phage components involved in interacting with bacterial receptors. Phage encoded receptor binding proteins (RBPs) mediate recognition and attachment to host cells during viral entry. The study of RBPs presents a promising avenue for discovering new antimicrobial candidates, engineering of phages to overcome bacterial resistance to phage, and informing the development of new therapeutic approaches and tools to overcome pathogen detection limits [15,16,17,18,19].

Although RBPs have different receptor targets, they possess similarities. These proteins are typically resistant to proteases and are thermostable. Additionally, they often assemble in trimeric states and feature conserved β-sheets in their central domains [16,20,21,22]. Classically, the N-terminal regions are responsible for binding to other virion components (i.e., baseplates) while the C-terminal regions interact with receptors on host cells [23]. Examples of RBPs include tail fibers, tail spikes, and head fibers [9,10,20,24]. Phages that use polysaccharides as receptors carry proteins with the ability to enzymatically degrade these sugar chains. These proteins are broadly defined as depolymerases and target glycan-containing bacterial components on host cell surfaces such as LPS, EPS, and CPS via their central domains [1,2,4,24,25]. The most well-studied depolymerase is Gp9 from *Salmonella* phage P22, which specifically targets LPS by cleaving bonds between rhamnose and galactose in the O-antigen [15,20,26]. Administration of purified Gp9 has been shown to reduce *Salmonella* loads in chickens [15]. The promise for depolymerase-based therapies has also been demonstrated in other systems highlighting their potential for use as therapeutics or adjuvants. Furthermore, their efficacy in degrading biofilms has sparked an increase in the number of CPS or LPS specific depolymerase structures available in the last decade [16,20,21,27,28,29,30,31,32,33,34,35,36,37,38,39].

Identification of RBPs has been largely conducted via bioinformatics analyses, which are dependent on sequence homology to well-characterized RBPs and/or genetic synteny [40]. This approach is problematic because it excludes the detection of RBPs that diverge from those found in well-studied systems. Often, RBPs remain unidentified in phage genomes or are instead annotated vaguely as “tail protein” or “baseplate protein”. Although such designations aid in narrowing down the search for RBPs within one genome, the experimental verification of RBP identity and function is required. This typically involves cloning, expression, and purification of the candidates. Inarguably, this is time consuming and labor intensive with no guarantee of identifying an RBP. The need for more rapid methods allowing the identification of novel RBPs has not gone unnoticed and one method to address this issue has already been developed [22].

The focus of this work was to uncover the identity of RBPs in *Agrobacterium* phage 7-7-1. This phage’s host range is limited to some *Agrobacterium* spp., which are gram negative bacteria most well-known for causing crown gall disease [23,41]. Phage 7-7-1 is a flagellotropic or flagella-dependent member of the *Myoviridae* family [23,41,42]. This class of phages begin infection by interacting and binding to the flagellar filaments of their bacterial hosts [12,40,41,42,43,44,45,46,47]. Flagella are used by bacteria to move throughout their environments and are powered by a motor located at the flagellar base that relies on proton motive force [48]. In *Alphaproteobacteria*, such as *Agrobacterium* spp., the flagellar motors only rotate clockwise and any alteration in swimming direction is achieved by modifying rotational speed of individual flagellar motors [49,50]. Recognition of flagella may confer another level of host specificity for these phages and provides a means to reach the cell surface, subsequently aiding interactions with other cell surface components. The mechanisms underlying the translocation of phages on flagellar filaments continues to be an open question and seems to lack a universal mechanism [12,43,44,51].

We have previously shown that infection by phage 7-7-1 is negatively affected by reductions in flagellar length and number and, most notably, that flagellar rotation is required for infection [42]. We also discovered that LPS is an essential secondary cell surface receptor [13]. Considering the different mechanisms employed by flagella-dependent phages during infection, we hypothesize that RBPs in these systems would be different than those currently described. To date, there has been no identification of an RBP in a flagellotropic phage. In this work, we set out to pinpoint RBPs in *Agrobacterium* phage 7-7-1. Ultimately, information gained from these studies will contribute to a model that delineates flagella-dependent phage entry mechanisms and informs possible protein-based therapeutics against *Agrobacterium* spp.

## 2. Materials and Methods

### 2.1. Bacterial Strains and Plasmids

*E*. *coli* and *Agrobacterium* sp. H13-3 [52] strains, their derivatives, and the plasmids used are listed in Table 1.

### 2.2. Media and Growth Conditions

*Agrobacterium* sp. H13-3 strains were grown in TYC (0.5% tryptone, 0.3% yeast extract, and 0.087% CaCl_2_ × 2 H_2_O (pH 7.0)) or NY (0.8% nutrient broth and 0.3% yeast extract) at 30 °C. *E. coli* strains were grown in lysogeny broth (LB) [57] at 37 °C. For *E. coli*, kanamycin was used at a concentration of 50 μg mL^−1^ and chloramphenicol at 30 μg mL^−1^ in LB. Streptomycin and neomycin were used at 600 μg mL^−1^ and 120 μg mL^−1^, respectively, in TYC or NY when culturing *Agrobacterium* sp. H13-3.

### 2.3. Phage Propagation and Purification

The protocol for propagating and purifying phage was followed as previously described [13,42]. Briefly, 200 mL NY cultures of *Agrobacterium* sp. H13-3 cells at an OD_600_ of 0.03 were infected with phage at an MOI of 0.005 and incubated with shaking at 30 °C for 24 h. NaCl was added to a final concentration of 4%, left on ice for 30 min, and centrifuged at 10,000× *g* for 30 min at 4 °C. Precipitation was accomplished by adding polyethylene glycol 8000 to the supernatant (10% *w*/*v*) and further incubation at 4 °C for 16 h. Phage particles were sedimented by centrifugation, suspended in 2 mL of TM buffer (20 mM Tris-HCl (pH 7.5) and 10 mM MgSO_4_), and overlaid on a 10% to 50% (*w*/*v*) iodixanol (OptiPrep; Accurate Chemical and Scientific Corporation, Westbury, NY, USA) gradient. Following centrifugation at 200,000× *g* for 2 h at 15 °C a blue-white band containing virions was extracted with an 18-gauge syringe and dialyzed against TM buffer at 4 °C. Phage was titered via the standard plaque assay. The phage titer ranged from 10^10^ to 10^12^ PFU mL^−1^. Phage stocks were stored long term at 4 °C.

### 2.4. Phage 7-7-1 DNA Library Construction

Phage DNA was extracted using the phenol-chloroform method. Briefly, 150 μL 5% sodium lauroyl sarcosinate and 4 μL proteinase K (100 mg/mL) were added to 1 mL of phage stock (1.87 × 10^11^ PFU). The mixture was incubated at 50 °C for 2 h and 100 μL of 3 M sodium acetate (pH 5.2) was added to the mixture. One volume of phenol:chloroform:isoamyl (25:24:1) was added, after which the sample was vortexed vigorously and centrifuged for 5 min at 14,000× *g* at room temperature. The aqueous phase containing DNA was removed and placed in a new microcentrifuge tube and the phenol-based separation was repeated two more times. DNA was precipitated by adding an equal volume of isopropanol followed by centrifugation. The DNA containing pellet was washed with 70% ethanol and suspended in 50 μL of nuclease-free water. Following purification, the phage DNA was randomly fragmented via nebulization. In brief, 5–25 μg of phage DNA was diluted in 750 μL of shearing buffer (10 mM Tris, 1 mM EDTA, and 10% glycerol (pH 8.0)). Random fragmentation of the phage DNA was achieved by nebulizing under 10 psi for 20 s, generating fragments from 1-4 kb. The desired range of fragment sizes was determined by analyzing the distribution of gene sizes in the phage 7-7-1 genome. Precipitation of DNA was conducted by adding 0.3 M sodium acetate (pH 4.8), 4 μL of 20 mg/mL of mussel glycogen (Roche, Mannheim, Germany), and 700 μL of isopropanol and incubating on dry ice for 15 min or at −20 °C overnight. The precipitated DNA was then centrifuged at 12,000× *g* for 15 min at room temperature, washed with 800 μL of 80% ethanol, and centrifuged again as before. Once the pellet was dry, it was suspended in nuclease-free water. The DNA fragments were blunt end repaired with T4 DNA polymerase (NEB, Ipswich, MA, USA) following the manufacturer’s protocol. The blunt end repaired fragments were precipitated as described above and mixed with dephosphorylated and EcoRV digested pET30a in preparation for ligation. The ligation reaction was set up in accordance with the manufacturer’s instructions using 50 ng of phage DNA and 20 ng of the vector. Transformation of *E. coli* TOP10 cells with the ligation mixture was achieved using heat shock. The presence of phage DNA fragments was evaluated by purifying plasmids and conducting single digestion reactions using EcoRV (NEB, Ipswich, MA, USA). We observed that 7 of 10 colonies contained pET30a with phage fragment inserts. Following confirmation, colonies were pooled, and the plasmid DNA was isolated using the Wizard Plus SV Miniprep system (Promega, Madison, WI, USA). BL21(DE3) cells were then transformed with this library of phage DNA. This process was repeated until we obtained the target number of transformants required for 1.7× coverage of the phage 7-7-1 genome. This number was determined by the Clarke–Carbon equation: N = ln (1−P)/ln (1−1/f) where P is the probability that a fragment will be present in the library and F is the average fragment size/genome size in bp.

### 2.5. RBP Screen

This protocol was followed as previously described [22] with some modifications. In summary, *E. coli* BL21 (DE3) cells harboring the phage 7-7-1 genomic library were grown on LB supplemented with kanamycin and lifted onto 82 mm diameter Whatman Protran^®^ nitrocellulose membranes. The membranes were then placed colony side up on LB supplemented with kanamycin and 0.4 mM β-D-1 thiogalactopyranoside (IPTG) and incubated overnight at 30 °C to allow for protein expression. Following induction, the membranes were removed from the agar, placed in empty petri dishes, and freeze/thawed five times at 5-min intervals. Circular, pre-cut Whatman filter paper was saturated with the commercially available bacterial protein extraction reagent (BPER, Thermo Scientific, Rockford, IL, USA) supplemented with 1 x HALT™ protease inhibitor (Thermo Scientific, Rockford, IL, USA), 3 mg of lysozyme, and DNase I (1 U/mL), and protein containing membranes were placed on top of the filter paper for 1 h at room temperature. The membranes were washed once with phosphate buffered saline (100 mM NaCl, 80 mM Na_2_HPO_4_, and 20 mM NaH_2_PO_4_ (pH 7.4)) with 0.5% Tween 20 (PBST) for 5 min, then blocked in PBST containing 5% skim milk for 1 h. Following blocking, membranes were washed with PBST three times for 5–10 min. Remaining colony debris was removed by lifting with a Kimwipe. Membranes were UV-irradiated for 15 min and then incubated in 500 mM NaCl at room temperature overnight on a slowly rotating platform. Following 3 washes with PBST as described above, a suspension containing 10^7^ CFU/mL in blocking solution was added to the membranes for 1 h at room temperature with gentle shaking. Membranes were washed 3 times with PBST and excess liquid removed by flicking the membranes. The membranes were then placed on TYC plates supplemented with streptomycin, incubated at 30 °C overnight, and then at room temperature for a maximum of 24 h. Host cell growth was compared to the original position of colonies on the master plate (from which the colonies were lifted). As a verification, these colonies were subjected to a second round of screening. Transformants capable of producing protein that bound host cells were cultured in LB at 37 °C overnight and plasmids isolated using the Wizard Plus SV Miniprep system (Promega, Madison, WI, USA). Finally, the plasmids were sequenced using the oligonucleotides T7 terminator primer (GCTAGTTATTGCTCAGCGG) or T7 promoter primer (TAATACGACTCACTATAGGG) to uncover the sequence of the phage genome fragment. For the confirmatory RBP screen, the same protocol was essentially followed except that the initial transformants contained the plasmids pBS1218 to pBS1228 (Table 1). For visualization, membranes were stained with amido black stain 10b (0.25% amido black, 50% methanol, and 10% acetic acid) for 5 min, and destained twice for 10 min with destaining solution (45% methanol and 10% acetic acid).

### 2.6. Genetic Construction of Recombinant Plasmids and Bacterial Strains

Phage 7-7-1 DNA was isolated as described above. Plasmid DNA was isolated using the Wizard Plus SV Miniprep system (Promega, Madison, WI, USA) while PCR products were purified from agarose gels using the Wizard SV gel and PCR clean-up kit (Promega, Madison, WI, USA). These amplicons were cloned into pET30a using restriction enzymes to obtain fusions with N- or C- terminal 6x histidine tags. Following Sanger sequencing to confirm the constructs, *E. coli* strains BL21(DE3) or Lemo 21(DE3) were transformed with appropriate plasmids using heat shock in preparation for protein expression. Allelic replacement was used to generate the *Agrobacterium* sp. H13-3 ∆*fliK* deletion strain as described previously [13,42].

### 2.7. Recombinant Protein Expression and Purification

Cells were grown at 37 °C in LB plus the appropriate antibiotic with shaking (225 rpm) until an OD_600_ of 0.6–0.8 was reached. IPTG was added to a final concentration of 0.4 mM and cultures grown at 30 °C for 5 h. Lemo21 (DE3) cells were used and 250 μM rhamnose was added to the LB medium before induction. Cells were harvested by centrifugation and suspended in binding buffer (20 mM NaP_i_, 500 mM NaCl, and 20 mM imidazole (pH 7.4)) supplemented with 1 mM phenylmethylsulfonyl fluoride (PMSF). Lysis was achieved by passing the cell suspension 3 times through a French pressure cell at 20,000 lbs/in^2^ (SLM Aminco, Silver Spring, MD, USA). Soluble and insoluble fractions were separated via centrifugation at 72,600× *g* at 12 °C for 45 min. The soluble lysates were filtered using a 0.2 μm polyethersulfone (PES) membrane syringe filter and loaded onto a 5 mL nickel-nitrilotriacetic acid (Ni-NTA) column (GE Healthcare Life Sciences) using fast-performance liquid chromatography (FPLC; ÄKTA Go; Cytiva Life Sciences, Marlborough, Massachusetts, USA). The protein was eluted in a linear gradient comprised of binding buffer and elution buffer (20 mM NaP_i_, 500 mM NaCl, and 400 mM Imidazole (pH 7.4)). Protein containing fractions were pooled and concentrated using an Amicon concentrator system and regenerated cellulose membranes (10 kDa MWCO; Millipore, Billerica, MA). The protein was dialyzed into PBS (100 mM NaCl, 80 mM Na_2_HPO_4_, and 20 mM NaH_2_PO_4_ (pH 7.2)). Visual analysis of purified protein was conducted via SDS-PAGE. Protein concentrations were determined by generating a standard curve using bovine serum albumin and Bradford reagent (Bio-rad, Hercules, California, USA) as described by the manufacturer and deducing the concentration of the target protein by mapping to the curve. Protein yields ranged from 0.02 mg/mL to 1 mg/mL from 4 L of expression cultures.

### 2.8. Liquid Clearance Assay

Stationary phase *Agrobacterium* sp. H13-3 cells were diluted to an OD_600_ of 0.03 in 4 mL of TYC containing streptomycin, and 0.6 μg/mL of purified Gp4_1-247_ was added to the culture. Density of the cultures was determined by measuring the OD_600_ following 24 h of incubation at 30 °C. Images of samples were taken with a Nikon D3400 camera.

### 2.9. Growth Curve Experiments

Stationary phase cultures of *Agrobacterium* sp. H13-3 in TYC with streptomycin were diluted to an OD_600_ of 0.03, and 200 μL were deposited in wells of clear flat bottom 96-well plates. Purified Gp4_1-247_ was added to each well at a concentration of 0.6 μg/mL and plates were sealed with AeraSeal™ breathable sealing film (Excel Scientific, Victorville, CA, USA). Plates were incubated in an INFORS HT Multitron at 30 °C for 48 h with shaking at 225 rpm. Cell densities were determined via OD_600_ at different time points using a BioTek Cytation 5 plate reader. As a positive control, phage 7-7-1 was added to at an MOI = 1. Equal volumes of PBS were added as negative controls.

### 2.10. Lawn Clearance Experiments

Bacterial strains were seeded in TYC top agar (0.5% agar) and overlaid on TYC nutrient plates. Following solidification of agar at room temperature, 10 μL of buffer, purified protein, or 10^7^ PFU of phage were spotted on the agar. Spots were allowed to dry completely at room temperature and then incubated at 30 °C overnight. Images of plates were captured using a Nikon D3400 camera.

## 3. Results

### 3.1. Construction of the Randomly Fragmented Phage 7-7-1 DNA Library and Identification of Candidate RBPs

**Figure 1:**
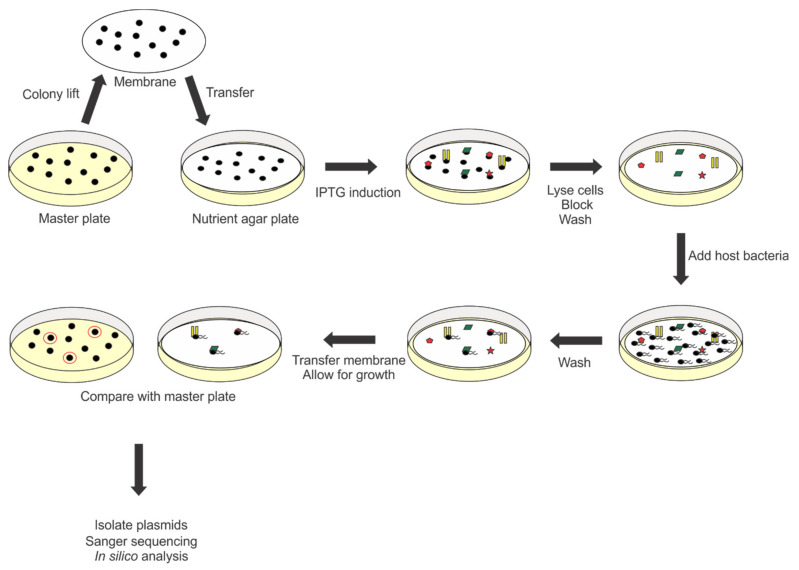
Illustration of RBP screening method. Transformants containing the phage genome library were lifted onto nitrocellulose membranes and protein expression was induced by placing on IPTG containing nutrient agar. Cells were lysed, membranes were blocked and washed prior to the addition of motile host cell bacteria. The membranes were washed to remove unbound host cells and then incubated at 30 °C to allow for host cell growth. Transformants containing RBP encoding gene fragments were identified by comparing areas of host cell growth on membranes with original locations on master plate. Plasmids were isolated and sequenced. In silico analysis of sequences was used to identify genes coding for RBPs and predicting ORFs.

**Figure 2:**
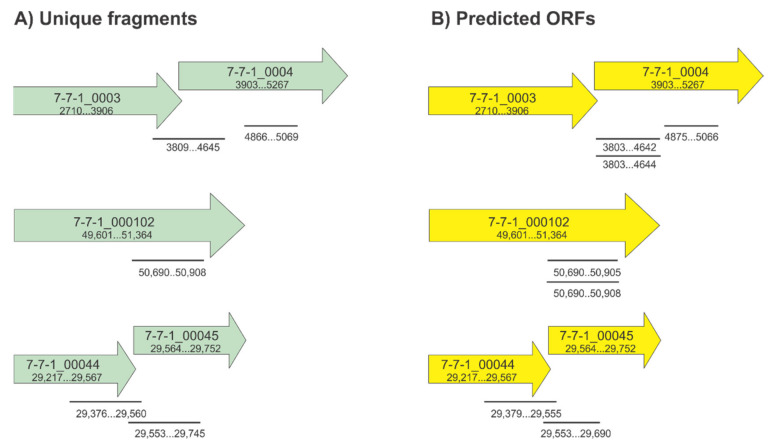
Fragment mapping to phage 7-7-1 genome. (**A**) illustration of unique fragments in reference to the genome and (**B**) predicted ORFs as determined by the SnapGene software.

An updated structural analysis of *Agrobacterium* phage 7-7-1 virions revealed the presence of head and tail filaments in addition to previously reported tail fibers (personal communication, Dr. Ariane Briegel, Leiden University). This finding indicated the possibility for the presence of multiple RBPs within phage 7-7-1 virions. However, the annotated genome contains only one gene with implied RBP function: *7-7-1_00102* [23]. We hypothesize that multiple RBP encoding genes were not identified bioinformatically because of their potential novel features. Therefore, we set out to discover RBPs in phage 7-7-1 using a previously developed method [22]. This involved generating a randomly fragmented genomic DNA library of phage 7-7-1 and screening this library for candidates using an RBP identification assay [22] (Figure 1). We screened 10,000 of these colonies and identified 13 clones capable of producing gene products that bound *Agrobacterium* sp. H13-3 cells. Upon purifying the plasmids and sequencing their inserts, we used the Basic Local Alignment Search Tool (http://blast.ncbi.nlm.nih.gov/Blast.cgi) to evaluate the identity of the phage genome fragments and the SnapGene software (from Insightful Science; available at snapgene.com) to analyze open reading frames (ORFs). We identified six unique fragments, spanning five genes (Table 2; Figure 2, left). By considering the predicted ORFs and predominantly encompassed genes per fragment, we further deduced which genes are more likely to code for RBPs. For example, the FG19-1 fragment is 753 bp in length and covers *7-7-1_0003* and *7-7-1_0004*. Only 16 bp map to *7-7-1_0003*, while 741 bp encompass *7-7-1_0004* (note that the reading frames are overlapping (Table 2, Figure 2)). Moreover, the other unique fragment mapping to this genomic region only encompasses *7-7-1_0004* (FG15, FG28; Table 2). Thus, the gene responsible for producing protein capable of binding host cells is *7-7-1_0004.* In a similar fashion, we pinpointed *7-7-1_00044* as the gene encoding a candidate RBP. One unique fragment includes only *7-7-1_00044* (FG5, F6) while the other fragment maps to *7-7-1_00044* and *7-7-1_00045* (FG42). Although the predicted 125 bp ORF for FG42 only covers 12 bp of *7-7-1_00044*, we deduced that this gene is likely the RBP encoding gene because of its recurrence in the screen. Meanwhile, fragments FG9, FG10, FG26, and FG44 only contain one gene, *7-7-1_000102.* Ultimately, we concluded that the genes responsible for the observed binding of host cells are *7-7-1_0004*, *7-7-1_000102*, and *7-7-1_00044.*

### 3.2. Predicted Functions of Gp4, Gp102, and Gp44

**Figure 3:**
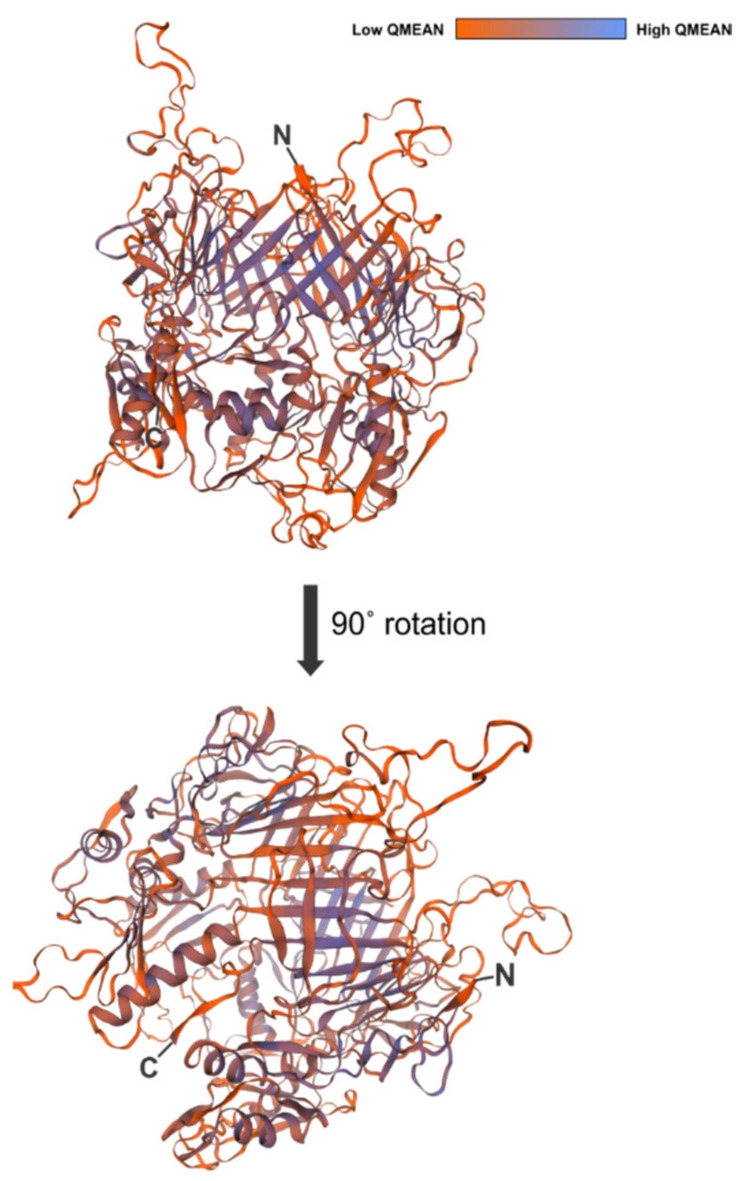
Predicted protein model of Gp4.SWISS-MODEL generated protein model for Gp4 with the absolute quality estimates (QMEAN) colorimetrically displayed. The prophage MuSo2 tail protein from *Shewanella oneidensis* (PDB ID 3CDD) was used as a template. The global model quality estimate (GMQE) was 0.46 while the sequence identity was 20.24%. The model encompassed 374/454 amino acids. N, N terminus; C, C terminus.

We then used BLASTP [58], Phyre2 [59], and SWISS-MODEL [60,61,62] to gain insight on possible protein functions and predicted structures of the gene products. According to BLASTP, Gp4 (*7-7-1_0004*) and Gp102 (*7-7-1_000102*) are associated with the phage tail, while Gp44 (*7-7-1_00044*) is a hypothetical protein (Table 3). Next, we used the Phyre2 server and SWISS-MODEL to create protein models based on homology and to gain further insight on potential function. The results produced using all three tools coincided. In BLASTP, Gp4 is annotated as a tail biosynthetic protein for its similarity to a Mu-like prophage tail protein in *Bradyrhizobium* sp. BTAi1 (YP_001242396.1). The top result with both Phyre2 and SWISS-MODEL is the phage MuSo2 tail protein from *Shewanella oneidensis* (PDB ID 3CDD). The Phyre2 generated model has 80% coverage, 21% sequence identity, and a confidence value of 100 (Table 3, top row). Using SWISS-MODEL we generated a homotrimeric homology model for Gp4 (Figure 3) with the prophage MusSo2 tail protein as a template. The global model quality estimate (GMQE) value is 0.46, and the sequence identity is 20%. The model spans 374 of 454 amino acids (82%), only lacking the last 80 amino acid residues. One noticeable feature in this model is the central β-barrel structure (Figure 3), which is a hallmark feature of depolymerases [16,21,22,26,30]. Thus, we hypothesize that Gp4 is an LPS targeting depolymerase for this phage.

We could not produce any high confidence models for Gp102 or Gp44 using Phyre2 or SWISS-MODEL. Either the coverage was very low with a high confidence value, or the coverage was high with lower confidence (Table 3, middle and bottom rows). Gp102 is designated as a putative tail fiber protein in BLASTP, due to its similarity to the large tail fiber proximal subunit in Enterobacteria phage JSE (YP_002922323.1). However, the top Phyre 2 template is the hydrolase XylC from *Thermoanaerobacterium* *saccharolyticum* JW/SL-YS485. The percent coverage is 9% with a 36% sequence identity and 96.3 confidence value. Gp44 was listed as a hypothetical protein in BLASTP. Using Phyre2, the top template is an aldolase from *Kordia algicida* OT-1 with a 37% coverage, 14% sequence identity, and a confidence value of 15. Similar results were obtained when using SWISS-MODEL (data not shown.). In conclusion, we present convincing evidence that Gp4 is associated with the phage tail but do not have straightforward insight into the predicted functions of Gp102 and Gp44.

### 3.3. Confirmation of Initial RBP Screen and Identification of Functional Plasmid Constructs

**Figure 4:**
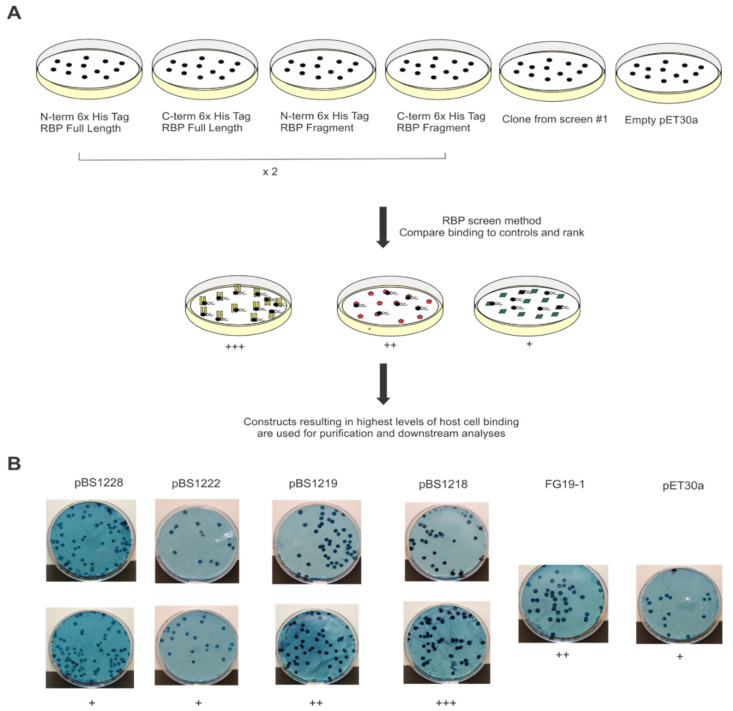
RBP confirmation screen summary. (**A**) transformants with known plasmid constructs were lifted onto nitrocellulose membranes and subjected to the RBP identification assay. Levels of host cell binding were phenotypically assessed compared to the controls (membranes containing empty pET30a or fragments identified in initial screen). (**B**) results following staining of membranes containing full length or truncated versions of Gp4 as well as positive and negative controls. Binding was graded as follows: (+++) specific binding in all or majority of areas containing protein, (++) binding in some areas containing protein, and (+) mostly nonspecific bacterial growth throughout the membrane.

To verify our previous findings and to create a strategy for subsequent analyses using purified proteins, we conducted a second, confirmatory RBP screen. This time, our starting *E. coli* populations contained defined plasmid constructs rather than the randomly fragmented phage 7-7-1 genome library. Specifically, plasmids contained the full length or truncated variants (reflecting the fragments of the genes identified in the initial screen) of the RBP encoding genes N- or C- terminally fused to a 6x histidine tag. Since the results from the initial screen only implicated fragments of the RBPs through this approach, we also sought to evaluate whether the full-length proteins are capable of binding host cells and, thus, which variants to use for subsequent approaches. *E. coli* cells containing vector pET30a were included as negative control and representative plasmids eliciting host binding from the initial screen as positive controls. To account for variations in binding, membranes containing proteins from *E. coli* cells with defined plasmid constructs were set up in duplicate. We developed a scoring system to better describe the observed levels of host cell binding. A schematic summarizing our observations and scoring is included in Figure 4 and Table 4, respectively.

To visualize binding, we stained the host cell containing membranes with the amido black 10b stain, which pigmented areas contained both protein and host cells (Figure 4B). A score of +++ reflects high levels of host cell binding in areas containing protein. Meanwhile, a ++ score represents host cell binding in some of the areas imbedded with protein. Finally, a score of + denotes non-specific binding as illustrated by bacterial growth throughout the membrane in few or no distinct spots. Results from stained membranes containing full length or truncated variants of Gp4 and controls are included in Figure 4B as a representative example of our scoring system.

During the confirmation screen for plasmids containing *7-7-1_0004*, we observed that the highest degree of host cell binding was mediated by proteins from *E. coli* BL21 (DE3) with pBS1218. This plasmid encodes a fragment of Gp4, encompassing amino acids 1-247 (Gp4_1-247_) N-terminally fused to a 6x histidine tag. Of the plasmids containing *7-7-1_000102,* proteins from *E. coli* BL21 (DE3) with pBS1221 elicited the most host cell binding in comparison to the positive and negative controls. This plasmid contains the full-length version of Gp102, C-terminally fused to a 6x histidine tag. Finally, *E. coli* BL21 (DE3) with pBS1224, encoding Gp44, C-terminally fused to a 6x histidine tag, was responsible for evoking the highest degree of host cell binding (Table 4). In summary, the second RBP screen confirmed that Gp4, Gp102, and Gp44 bound host cells. For the remaining part of this study, we focused on characterizing interactions between Gp4_1-247_ and *Agrobacterium* sp. H 13-3 host cells. This RBP candidate occurred with the highest incidence in the initial RBP screen and in silico analyses permitted the hypothesis that it is an LPS-targeting RBP of phage 7-7-1. Thus, we decided to investigate its activity in more detail.

### 3.4. Purified Gp4_1-247_ Inhibits the Growth of Host Cells in Liquid Cultures

**Figure 5:**
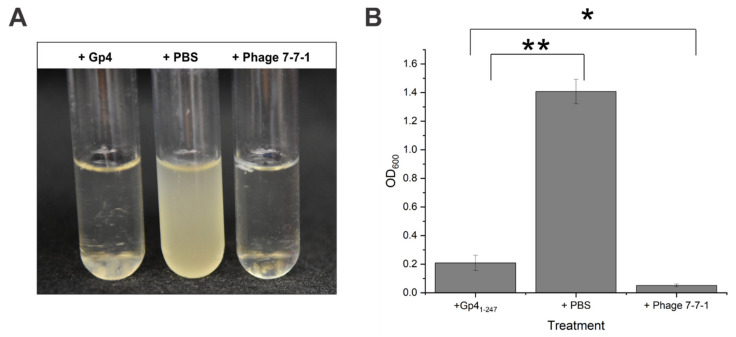
Effect of purified Gp4 on liquid cultures. Liquid cultures of *Agrobacterium* sp. H13-3 (OD_600_ = 0.03) received purified 40 μL Gp4 (0.06 μg/μL), PBS, or phage 7-7-1 (MOI = 1) and incubated at 30 °C for 24 h. Representative images of the cultures are shown in (**A**) while the final densities are illustrated in (**B**). Data reflects 3 biological replicates conducted in triplicate. The error bars represent standard deviations for each data set (student’s *t* test for Gp4_1-247_ and phage 7-7-1 treatments, *p* < 0.05; student’s *t* test for Gp4_1-247_ and PBS treatments, *p* < 0.01). The “*” denotes a p-value below 0.05 while the “**” indicates a *p*-value below 0.01.

We isolated Gp4_1-247_ with an N-terminal 6x histidine tag, produced from *E. coli* Lemo21 (DE3) cells transformed with pBS1218 (Table 1), via Ni-NTA affinity chromatography. Plasmid pBS1218 was selected because it encoded a protein that elicited the highest level of host cell binding during our confirmatory RBP screen described above. The Lemo21 (DE3) expression strain was chosen, because it allowed us to increase the proportion of soluble Gp4_1-247_ as has been documented for other proteins [63]. We then added the purified protein to growing bacterial cultures of wild-type *Agrobacterium* sp. H13-3. We observed a dramatic growth inhibition in liquid cultures as indicated by a seven-fold reduction in liquid culture turbidity following addition of Gp4_1-247_ compared to the control culture (Figure 5). The average OD_600_ for cultures receiving Gp4_1-247_ was 0.2, while for control cultures the average OD_600_ was 1.4. Meanwhile the density of cultures treated with phage 7-7-1 only reached an OD_600_ of 0.05. Visually, the clearance resulting from the addition of Gp4_1-247_ resembled that of control cultures receiving phage 7-7-1 (MOI = 1) (Figure 5A). However, this effect was not as pronounced, because the optical density of Gp4_1-247_ containing cultures was significantly higher than that of cultures supplemented with phage (Figure 5B; student’s *t* test, *p* < 0.05). We noticed sedimentation at the bottom of test tubes with cultures that received Gp4_1-247_, which was not observed with the other treatments (Figure 5A, left tube). The identity of this sediment and reasons underlying its presence are the subject of future investigations.

**Figure 6:**
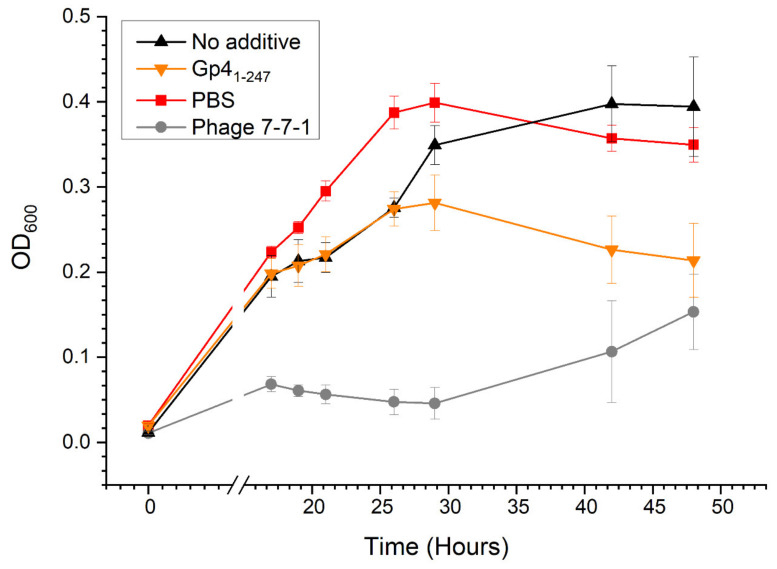
Growth curves for bacterial cultures under various conditions. *Agrobacterium* sp. H13-3 (OD_600_ = 0.03) cells were deposited in 96-well plates and received 2 μL of Gp4_1-247_ (0.06 μg/μL), PBS, phage 7-7-1 (MOI = 1), or no additive. Growth of the cultures was measured via OD_600_ at 17, 19, 21, 27, 29, 42, and 48 h using a plate reader. Data are representative of 4 independent experiments conducted in quadruplicate and error bars reflective of standard deviation. Student’s *t* test for Gp4_1-247_ and PBS or phage 7-7-1 at 42 and 48 h, *p* < 0.005.

To determine the effect of this protein on host cell growth, we generated growth curves of cultures in 96-well plates following addition of Gp4_1-247_, PBS, or phage 7-7-1 at an MOI = 1. We measured the density of these cultures at 0, 17, 19, 21, 26, 29, 42, and 48 h of growth. Treatment with Gp4_1-247_ resulted in growth stagnation at an OD_600_ of 0.27–0.28 from 26 to 29 h, followed by a slight decrease to an OD_600_ of 0.21 after 48 h of growth (Figure 6). The cultures that received PBS increased in growth for 29 h to a maximum OD_600_ of 0.40 and then experienced a decrease in growth at 42 and 48 h to an OD_600_ of 0.35. Meanwhile, the cultures without additive continued to increase in growth for 42 h to a maximum OD_600_ of 0.40 after which the growth plateaued. Compared to cultures without additive, Gp4_1-247_ treated cultures exhibited nearly a two-fold decrease in growth after 48 h (student’s *t* test; *p* < 0.005). In comparison, cultures with PBS grew to an OD_600_ that was 1.5 times higher than that of the Gp4_1-247_ treated cultures following 48 h of growth (student’s *t* test; *p* < 0.005). Cell cultures treated with phage 7-7-1 sustained at an OD_600_ below 0.01 until 29 h and then steadily increased to meet the density of the Gp4_1-247_ treated cultures at 42 and 48 h. This effect most likely reflects the emergence of phage resistant bacteria. Altogether, these results established that the liquid clearing effect by Gp4_1-247_ is likely bacteriostatic.

### 3.5. Lawn Clearance Effects of Purified Gp4_1-247_

**Figure 7:**
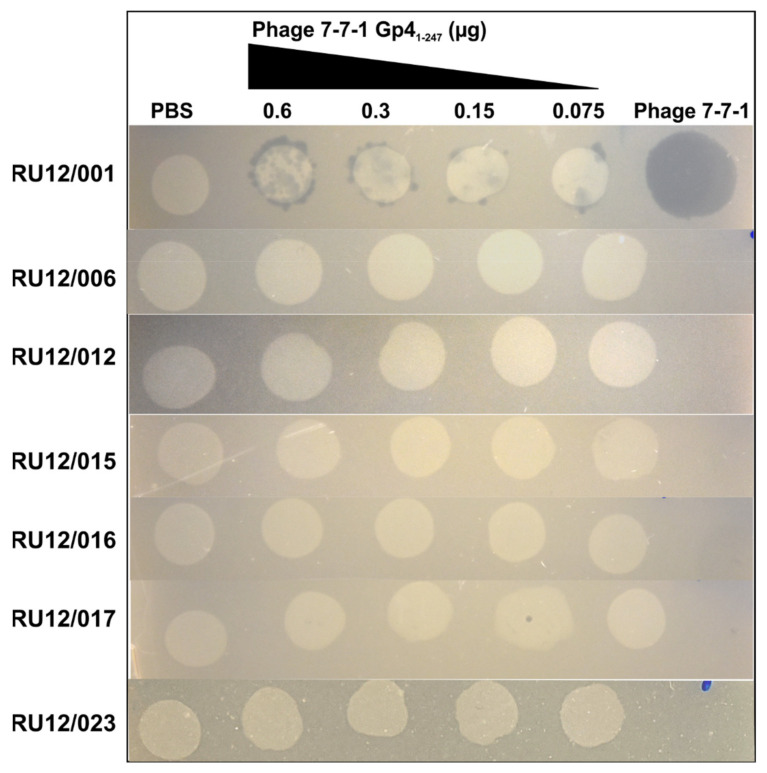
Lawn clearance experiments with Gp4_1-247_. TYC plates overlaid with TYC top agar (0.5% agar) containing wild-type and various phage resistant mutant strains received 10 μL spots of PBS, different concentrations of Gp4_1-247_, or phage 7-7-1 (10^7^ PFU). Following overnight incubation at 30 °C the plates were assessed for spots of clearance and imaged. The genotypes and resulting phenotypes for each strain are listed in Table 5.

To gain insight on factors mediating the observed bacteriostatic effect and to identify the receptor targets for this RBP, we evaluated the ability of purified Gp4_1-247_ to clear lawns of wild-type and phage-resistant *Agrobacterium* sp. H13-3 mutant strains. This was achieved by seeding bacteria grown under motile conditions (OD_600_ of 0.3) in top agar, overlayed on TYC plates containing streptomycin, which were then spotted with serial dilutions of Gp4_1-247_. Equal volumes of buffer and phage 7-7-1 were spotted as controls on the same plates. Following overnight incubation at 30 °C, we observed clearance of wild-type *Agrobacterium* sp. H13-3 cells in a dose-dependent manner on locations where Gp4_1-247_ dilutions were spotted. There was no evident lawn clearing in areas that received PBS, while spots of phage 7-7-1 produced uniform clearing (Figure 7; Table 5). Next, we evaluated whether Gp4_1-247_ could clear lawns of motility mutants that lack flagella (RU12/006) or possess non-rotating flagella (RU12/012 and RU12/023). These strains were resistant to clearance by Gp4_1-247_. This suggests that the activity of Gp4 is dependent on flagellar-mediated motility. We also conducted the same experiment on strains containing LPS mutations (RU12/015, RU12/016, and RU12/017) and observed the same resistance phenotypes to Gp4_1-247_. It is important to note that the motility of the LPS mutants does not differ from that of the wild-type strain (Table 5) [13]. Thus, these results suggest that the bacteriostatic activity of Gp4_1-247_ is dependent on actively rotating flagella and native LPS.

## 4. Discussion

Host recognition by bacteriophages is an important and essential step in initiation of infection. Initial interactions are mediated by phage encoded RBPs. Flagellotropic phages first bind to the bacterial flagellar filament and have been shown to interact with secondary cell surface receptors such as LPS, efflux pumps, and CPS [13,14,40,47]. RBPs in non-flagellotropic phages have been identified, resulting in a wealth of knowledge surrounding common features of these proteins including resistance to proteolysis, thermostability, and the ability to degrade glycans [16,20,21,24,28,29,30,31,32,33,34,35,36,37,38,39,64]. In myoviruses and siphoviruses, RBPs typically come in the form of tail fibers, while in podoviruses tail spikes are more common RBPs [9,10]. However, some myoviruses also contain tail fibers and tail spikes [21]. In addition, phages can possess head fibers that interact with host cell components [12,65]. The identity of these RBPs has largely relied on in silico analyses based on sequence and structure homology or genomic location [40]. However, for phages infecting non-model organisms, this approach can be less fruitful. In flagellotropic phages, the identification of RBPs has largely involved the visualization of head or tail filaments using electron microscopy (EM). Examples include the single long tail filament of phage χ, the head filament of φCbK, and three helical tail filaments of phage PBS1 [12,43,46]. When these flagellotropic phages were imaged together with their flagellated hosts, the filamentous RBPs were seen in close proximity to flagella, which is indicative of interaction. However, there are no published studies that provide proof for physical interaction, molecular binding mechanisms, or the genetic identity of these flagellotropic phage RBPs.

It is not unusual for phages to contain multiple RBPs as has been shown with myovirus CBA120. This phage has four tail spikes, each with different LPS targets that confer different host specificities allowing for a broader host range [64]. An updated structural analysis of phage 7-7-1 virions using cryogenic EM (cryo-EM) revealed the presence of head fibers and tail filaments in addition to previously observed bushy, short tail fibers (personal communication; Dr. Ariane Briegel, Leiden University) [23]. Thus, these appendages are prime RBP candidates, although their interactions with host flagella may not be as readily discernable in EM studies as those for phage χ, φCbK, or PBS1 [12,43,46]. Initially, it was postulated that the tail fibers were responsible for mediating interactions between phage 7-7-1 and host flagella, but this hypothesis has not been tested [41]. Although a “nut and bolt” model for the translocation of bacteriophage χ and other flagella-dependent phages has been proposed, it becomes increasingly clear that there is no uniform mode of phage translocation along the flagellar filament [12,45,51]. This is not only a result of the differences in phage appendages (i.e., head versus tail fibers), but also due to the different modes of flagellar rotation and filament structure. In both, *E. coli* and *C. crescentus*, the flagellar motors exhibit bidirectional rotation to switch between smooth swimming and tumbling behaviors as the cell moves through the environment in response to chemotactic stimuli [12,66]. Meanwhile, in *Agrobacterium* spp. and *Sinorhizobium meliloti*, the motors only exhibit rotation in clockwise direction and asynchronously slow down rotational speed of individual motors to switch between swimming and tumbling [49,50]. Additionally, *E. coli* has plain flagella, which switch helicity (left-handed to right-handed) and have a smooth surface structure. Meanwhile, *Agrobacterium* spp. have complex filaments that do not switch handedness and have a coarser surface structure [67,68]. Due to the differences in flagellar rotation and filament surface structure, the modes for phage translocation along the flagellar filament are likely different for phage 7-7-1 than for those that have been documented for phage χ and φCbK.

To gain more insight into flagellotropic phage interactions with their bacterial hosts and cognate receptors we decided to search for RBPs in phage 7-7-1. We screened a randomly fragmented genome library of phage 7-7-1 for RBP candidates using an in vivo host cell binding assay [22] (Figure 1). We identified six unique gene fragments that produced proteins responsible for binding *Agrobacterium* sp. H13-3 cells (Figure 2). The results implicated three genes encoding RBPs: *7-7-1_0004* (Gp4), *7-7-1_000102* (Gp102), and *7-7-1_0044* (Gp44) (Table 2). Fragments containing *7-7-1_0004* had the highest rate of occurrence (six fragments), followed by *7-7-1_000102* (four fragments) and *7-7-1-00044* (three fragments). A second screen with defined plasmid constructs confirmed the initial results (Table 4). In silico protein homology searches with Phyre2 implicated Gp4 as a tail associated protein, while the predicted functions of Gp102 and Gp44 were of a hydrolase and aldolase, respectively (Table 3). Although we were unable to produce high confidence structural models for Gp102 and Gp44, we did generate a model of Gp4 using SWISS-MODEL (Figure 3). This model was built using the structure of prophage MuSo2 tail protein from *S. oneidensis* as a template. Most notably, the model predicts the association of Gp4 in a homotrimeric state and the formation of a central β-barrel structure. Phage-derived depolymerases targeting LPS or CPS commonly possess β-sheets in their central domains, which contain the catalytic site responsible for enzymatic degradation of glycans and frequently form homotrimers [9,20,21,26,35]. We hypothesized that Gp4 functions as a depolymerase degrading the secondary cell surface receptor for phage 7-7-1: LPS. For these reasons, we focused on the further analysis of Gp4.

The confirmatory RBP binding screen identified the Gp4 fragment comprising amino acid residues 1-247 with an N-terminal 6x histidine tag as the most efficient at binding host cells (Table 4). Therefore, Gp4_1-247_ was purified using Ni-NTA affinity chromatography, and its effect on growing liquid cultures was evaluated. Interestingly, we discovered that Gp4_1-247_ inhibits the growth of host cultures in liquid and in lawns on agar surfaces (Figure 5, Figure 6 and Figure 7). Similar growth inhibition effects have been documented for another flagellotropic phage-encoded protein named FlaGrab. In this case, the protein’s activity is bacteriostatic as evidenced by the lack of lysed cells or debris in areas where host cell growth was cleared on agar plates [69,70]. Further investigations of mutant *Agrobacterium* sp. H13-3 strains indicated that growth inhibition is dependent on intact flagellation and flagellar rotation as well as on wild-type surface glycans (Figure 7). The FlaGrab protein of phage NCTC 12673 also inhibits the growth of its *Campylobacter jejuni* host in a motility dependent manner. However, in depth studies have demonstrated that this protein interacts with the flagellar filament via specific flagellar glycans [47,69]. It reduces motility of the host and alters the transcription of energy metabolism pathways [47,65,66]. Initial motility assays on soft agar swim plates seeded with Gp4_1-247_ did not result in a significant reduction in motility. However, when Gp4_1-247_ was pre-mixed with wild type *Agrobacterium* sp. H13-3, swim ring geometries exhibited an irregular shape, suggesting some type of interference (data not shown). The interaction of Gp4 with host flagellar filaments will be the subject of future studies.

FlaGrab was initially thought to function as an RBP, but its absence in virions suggested that it may instead function as an effector protein [69]. Similar to Gp4, flagellar rotation is also required for the interaction of FlaGrab with its host. CPS is an additional receptor for phage NCTC 12673 and mutations to CPS result in phage resistance. However, FlaGrab is still able to clear lawns of CPS mutants, indicating that its activity is not dependent on CPS hydrolysis. Meanwhile, Gp4_1-247_ is not able to clear lawns of non-motile mutants that lack flagella or possess non-rotating flagella. LPS mutants are also resistant to Gp4_1-247_ mediated growth inhibition, indicating a reliance on flagellar function and wild-type LPS (Figure 7). Thus, although FlaGrab and Gp4 possess some mechanistic similarities, there are also distinct differences in their mode of action.

The mechanism underlying the observed growth inhibition by FlaGrab was not determined. However, a mechanosensing hypothesis was proposed, in which FlaGrab binding to flagella reduces motility by causing flagellar stiffness. The cell then compensates by increasing proton flow to power flagellar rotation, which disrupts the electron transport chain and alters transcription of energy metabolism pathways. Although the proposed mechanosensing hypothesis could arguably occur in response to any protein that binds to flagella, FlaGrab relies on the specific recognition of flagellar glycans, which are present on the central, surface exposed regions of flagellar filaments [71]. Thus, the proposed response would only occur if this protein was also able to use flagellar glycans for the recognition of and binding to flagella.

Flagellar glycosylation affects flagellar function and assembly in different bacterial species. In *C. jejuni*, absence of flagellar glycosylation precludes successful assembly of functional flagella. In *C. crescentus,* flagellar filament assembly is also prevented when flagellar glycosylation is abolished and is likely due to a deficiency in export or reduced stability of non-glycosylated flagellin. Precursors of LPS, CPS, and peptidoglycan are used for the synthesis of flagellar glycans, highlighting a link between these important sugar containing structures and flagellar glycosylation [71]. According to our preliminary data, *Agrobacterium* sp. H13-3 possesses glycosylated flagellar filaments (data not shown). Although we have not yet determined the specific flagellin glycosylation patterns, future studies will investigate whether Gp4 activity is also dependent on the presence of specific flagellar glycans. This information will allow us to learn more about the role of flagellar glycosylation in *Agrobacterium* spp., which currently remains an unexplored research question, and to compare FlaGrab and Gp4 modes of action in further detail. Since flagellar glycosylation has been shown to play an important role in flagellar assembly and function in a variety of bacteria, we hypothesize that this function is conserved in *Agrobacterium* spp. and that it influences flagellotropic phage infection.

Currently, our data do not warrant the establishment of a working hypothesis for the mechanism underlying Gp4 mediated growth inhibition of *Agrobacterium* sp. H13-3. However, we can conclude that the host cell growth inhibition is dependent on functional flagella and native LPS. Although the role of RBP mediated host cell growth inhibition remains to be investigated it is possible that this induced growth retardation allows for efficient rerouting of cellular metabolic pathways towards phage propagation. However, it is possible that inhibiting the growth of the bacterial hosts allows time for the necessary metabolic restructuring The presence of phage proteins that are reliant on functional flagellar filaments and presence of specific glycans in two different flagellotropic phages is intriguing and warrants further investigation. The existence of these proteins with similar function proposes that FlaGrab-like proteins play an important and conserved role in flagellotropic phage infection processes. Uncovering the molecular mechanisms behind these interactions will aid in a more comprehensive model for flagella-dependent phage infections.

## Figures and Tables

**Table 1:** Strains and plasmids.

Strain/Plasmid	Relevant Characteristics ^a^	Source or Reference
Strain		
*E. coli*		
BL21 (DE3)	F^−^ *ompT hsdS_B_* (r_B_^−^ m_B_^−^) *gal dcm* λ (DE3)	Novagen
DH5α	*recA1 endA1*	[53]
Lemo21 (DE3)	*fhuA2 [lon] ompT gal (λ DE3) [dcm] ∆hsdS/ pLemo*(Cm^r^) pLemo *=* pACYC184*-PrhaBAD-lysY*	NEB
S17-1	*recA endA thi hsdR* RP4-2 Tc::Mu::Tn7 Tp^r^ Sm^r^	[54]
TOP10	F^-^ *mcrA* Δ(*mrr-hsdRMS-mc*rBC) *Φ80lacZ*ΔM15 Δ *lacX74 recA1 araD139* Δ*(araleu)*7697 *galU galK rpsL* (Sm^r^) *endA1 nupG*	Gift from Earl Petzold
*Agrobacterium* sp. H13-3		
RU12/001	Sm^r^; spontaneous streptomycin-resistant wild-type strain	[55]
RU12/006	Sm^r^; Δ*flaA*, Δ*flaB*, Δ*flaD*	[55]
RU12/012	Sm^r^; Δ*motA*	[42]
RU12/015	Sm^r^; Δ*AGROH133_08824*	This study
RU12/016	Sm^r^; Δ*AGROH133_07337*	This study
RU12/017	Sm^r^; Δ*AGROH133_13050*	This study
RU12/023	Sm^r^; Δ*fliK*	This study
Plasmid		
pET30a	Km^r^, expression vector (N- or C- terminal 6x histidine tag)	Gift from Christine Szymanski
pBS1218	Km^r^, *7-7-1_0004* fragment (741 bp) cloned into pET30a restriction sites NcoI/XhoI with N-terminal 6x histidine tag	This study
pBS1219	Km^r^, *7-7-1_0004* fragment (741 bp) cloned into pET30a restriction sites BamHI/SacI with C-terminal 6x histidine tag	This study
pBS1220	Km^r^, full length *7-7-1_000102* (1758 bp) cloned into pET30a restriction sites NcoI/BamHI with N-terminal 6x histidine tag	This study
pBS1221	Km^r^, full length *7-7-1_000102* (1761 bp) cloned into pET30a restriction sites NcoI/XhoI with C-terminal 6x histidine tag	This study
pBS1222	Km^r^, full length *7-7-1_0004* (1362 bp) cloned into pET30a restriction sites BamHI/SacI with N-terminal 6x histidine tag	This study
pBS1223	Km^r^, full length *7-7-1_00044* (348 bp) cloned into pET30a restriction sites BamHI/SacI with N-terminal 6x histidine tag	This study
pBS1224	Km^r^, full length *7-7-1_00044* (348 bp) cloned into pET30a restriction sites SalI/XhoI with C-terminal 6x histidine tag	This study
pBS1225	Km^r^, *7-7-1_00044* fragment (189 bp) cloned into pET30a restriction sites BamHI/SacI with N-terminal 6x histidine tag	This study
pBS1226	Km^r^, *7-7-1_00044* fragment (189 bp) cloned into pET30a restriction sites SalI/XhoI with C-terminal 6x histidine tag	This study
pBS1227	Km^r^, *7-7-1_000102* fragment (222 bp) cloned into pET30a restriction sites NcoI/XhoI with C-terminal 6x histidine tag	This study
pBS1228	Km^r^, full length *7-7-1_0004* cloned into pET30a restriction sites NcoI/XhoI with C-terminal 6x histidine tag	This study
pK18*mobsacB*	Km^r^; *lacZ mob sacB*	[56]

^a^ Cm^r^, chloramphenicol resistance; Sm^r^, streptomycin resistant; Km^r^, kanamycin resistant; bp, base pairs.

**Table 2:** Mapping of DNA fragments to phage 7-7-1 genome.

Gene and Location	ID	Fragment Range (bp)	# of Unique Fragments	Coverage per Gene (bp)	Snapgene Predicted ORF Range	N or C Terminal His Tag
7-7-1_0003 (2710…3906) 7-7-1_0004 (3903…5267)	FG7 FG8 FG15 FG19-1 FG28 FG89	3809…4645 3809…4645 4866…5069 3809…4645 3809…4645 3809…4645	2	16/1197 (03)	3903…4644 3903…4642 4875…5066 3903…4642 4875…5066 3903…4642	C C N C N C
				741/1365 (04)		

				or		


				204/1365 (04)		
7-7-1_000102 (49,601…51,634)	FG9	50,690…50,908	2	219/1764 (102)	50,693…50,905	N
	FG10	50,690…50,908			50,693…50,905	N
	FG26	50,690…50,908			50,690…50,908	N
		46,134…46,017				
	FG44	50,690…50,908			50,693…50,905	N
7-7-1_00044 (29,217…29,567) 7-7-1_00045 (29,564…29,752)	FG5 FG6 FG42	29,376…29,560 29,376…29,560 29,553…29,745	2	185/351 (44)	29,379…29,555 29,379…29,555 29,553…29,690	N N N

				or		

				14/351 (44)		
				127/189 (45)		

**Table 3:** RBP candidate protein homology.

Protein	BLASTP Function	Phyre2				
		Top Template	Amino Acids Aligned	% Coverage	% Identity	Confidence Value
Gp4 (454 AA)	Tail biosynthetic protein	Prophage MuSo2 tail protein from *Shewanella oneidensis*	2-366	80	21	100
Gp102 (587 AA)	Putative tail fiber	Hydrolase XylC from *Thermoanaerobacterium saccharolyticum* JW/SL-YS485	192-248	9	36	96.3
Gp44 (116 AA)	Hypothetical protein	Aldolase from *Kordia algicida* OT-1	65-109	37	14	15

**Table 4:** Confirmatory RBP screen binding analysis.

Gene	Plasmids	Protein Expressed	Binding *^c^*
*7-7-1_0004*	pBS1218	6xHis-Gp4_1-247_	+++
	pBS1219	Gp4_1-247_-6xHis	++
	pBS1222	6xHis-Gp4	+
	pBS1228	Gp4-6xHis	+
	FG19-1	Gp4_1-246_-6xHis *^a^*	++
	pET30a	---	+
*7-7-1_000102*	pBS1220	6xHis-Gp102	+
	pBS1221	Gp102-6xHis	++
	pBS1227	Gp102_363-436_-6xHis	+
	FG9	6xHis-Gp102_363-436_	+++
	pET30a	---	+
*7-7-1_00044*	pBS1223	6xHis-Gp44	+
	pBS1224	Gp44-6xHis	+++
	pBS1225	6xHis-Gp44_53-115_	++
	pBS1226	Gp44_53-115_-6xHis	+
	FG5	6xHis-Gp44_53-114_ *^b^*	+++
	pET30a	----	+

*^a^* Fragment ended with two nucleotides that were not in frame; therefore, pBS1218 and pBS1219 include an extra nucleotide to generate a fragment with an additional native codon. *^b^* Fragment ended with two nucleotides that were not in frame; therefore, pBS1225 and pBS1226 include an extra nucleotide to generate a fragment with an additional native codon. *^c^* (+++) denotes specific binding in areas containing protein, (++) indicates mostly specific binding with some background growth, and (+) represents nonspecific bacterial growth throughout the membrane (background).

**Table 5:** *Agrobacterium* sp. H13-3 strain genotypes and resulting flagellar and motility associated properties.

Strain	Genotype	Flagellar or Motility Phenotype	Other Phenotypes
RU12/001	Wild type	Normal motility	n/a
RU12/006	Δ*flaA* Δ*flaB* Δ*flaD*	No flagella	n/a
RU12/012	Δ*motA*	Non-motile	n/a
RU12/015	ΔAGROH133_08824	Normal motility	Mutant LPS
RU12/016	ΔAGROH133_07337	Normal motility	Mutant LPS
RU12/017	ΔAGROH133_13050	Normal motility	Mutant LPS
RU12/023	Δ*fliK*	Non-motile; abnormal hook	n/a

## Data Availability

All data are available from the corresponding author upon request.
